# Time of day differences in the regulation of glutathione levels in the rat lens

**DOI:** 10.3389/fopht.2024.1407582

**Published:** 2024-08-15

**Authors:** Bo Li, Haruna Suzuki-Kerr, Renita M. Martis, Christopher J. J. Lim, Zhou-ai Wang, Tai X. Nguyen, Paul J. Donaldson, Raewyn C. Poulsen, Julie C. Lim

**Affiliations:** ^1^ Department of Physiology, School of Medical Sciences, University of Auckland, Auckland, New Zealand; ^2^ New Zealand National Eye Centre, University of Auckland, Auckland, New Zealand; ^3^ Department of Pharmacology, University of Auckland, Auckland, New Zealand

**Keywords:** lens, circadian clock, glutathione, antioxidant, NRF2

## Abstract

**Introduction:**

Evidence in non-ocular tissues indicate that the antioxidant glutathione (GSH) may be regulated in a circadian manner leading to the idea that GSH levels in the lens may also be controlled in a circadian manner to anticipate periods of oxidative stress.

**Methods:**

Male rat Wistar lenses (6 weeks) were collected every 4 hours over a 24-hour period at 6am, 10am, 2pm, 6pm, 10pm and 2am and quantitative-PCR, western blotting and immunohistochemistry performed to examine the expression of core clock genes and proteins (BMAL1, CLOCK, CRY1-2, PER 1-3) and their subcellular localisation over a 24-hour period. Western blotting of lenses was also performed to examine the expression of NRF2, a transcription factor involved in regulating genes involved in GSH homeostasis and GSH related enzymes (GCLC, GS and GR) over the 24-hour period. Finally, HLPC was used to measure GSH levels in the aqueous humour and lenses every 4 hours over a 24-hour period.

**Results:**

The rat lens contains the core molecular components of a circadian clock with the expression of core clock proteins, NRF2 and GSH related enzymes fluctuating over a 24-hour period. BMAL1 expression was highest during the day, with BMAL1 localised to the nuclei at 10am. NRF2 expression remained constant over the 24-hour period, although appeared to move in and out of the nuclei every 4 hours. GSH related enzyme expression tended to peak at the start of night which correlated with high levels of GSH in the lens and lower levels of GSH in the aqueous humour.

**Conclusion:**

The lens contains the key components of a circadian clock, and time-of-day differences exist in the expression of GSH and GSH related enzymes involved in maintaining GSH homeostasis. GSH levels in the rat lens were highest at the start of night which represents the active phase of the rat when high GSH levels may be required to counteract oxidative stress induced by cellular metabolism. Future work to directly link the clock to regulation of GSH levels in the lens will be important in determining whether the clock can be used to help restore GSH levels in the lens.

## 1 Introduction

The lens contains high levels of the antioxidant glutathione (GSH) which exceed levels found in other ocular tissues (1, 2). These high levels are important in protecting the lens from oxidative stress and maintaining lens transparency. However, with advancing age, GSH levels decrease initiating a series of events such as loss of protein thiols, an increased in mixed disulfides, an increase in insoluble protein, protein aggregation and ultimately cataract formation (3–6). In the young lens, high GSH levels are maintained by several different pathways including the direct uptake of GSH from the ocular humours, intracellular synthesis of GSH from cysteine, glutamate and glycine by the sequential actions of the enzymes glutamate cysteine ligase (GCL) and glutathione synthetase (GS), regeneration of GSH from oxidised GSH (GSSG) by GSH reductase (GR), and export and degradation of GSH into its precursor amino acids for re-uptake by the lens (7). However, little is known about how GSH levels in the lens are regulated.

Circadian clocks in the body are self-sustaining endogenous oscillators that possess a timekeeper function to generate circadian rhythms which drives mammalian physiological and behavioural processes within a 24- hour cycle (8). Circadian rhythms are generated by the rhythmic expression of clock genes that involve transcriptional-translational feedback loop (9). These loops have a positive arm (BMAL1 and CLOCK) and a negative arm (PER1–3, CRY1&2). BMAL1 and CLOCK form a complex in the nucleus that bind to target gene promoters, resulting in the initiation of transcription of specific genes including genes of the negative arm of the clock. As the negative arm, PER and CRY proteins heterodimerise to repress the transcription of BMAL1 and CLOCK, with the BMAL1/CLOCK and PER/CRY cycle taking ~24 hours. Emerging evidence in non-ocular tissues suggests a connection between antioxidant balance and the circadian clock with antioxidants and antioxidant enzymes displaying daily cycles in their expression or activity levels (10, 11). Previous studies have reported daily fluctuations in levels of GSH in the mouse liver (12, 13), human platelets (14), mouse pancreas (15), and rat cerebral cortex (16). Enzymes involved in the synthesis of GSH, such as GCL and GR also exhibited rhythms in their mRNA expression and activity in rodent tissues (13, 17, 18). However, there is no clear pattern as to when GSH levels or enzyme expression/activity peak or trough between diurnal versus nocturnal animals or between rat and mouse suggesting it may be tissue specific. Time of day differences in GSH levels in *Drosophila* were shown to be controlled by a circadian clock as loss of Cycle (CYC; BMAL1 in mammals) or PER resulted in loss of temporal GSH fluctuations (19). Moreover, mRNA expression and activity of GCL, also displayed circadian rhythms, which were lost with loss of CYC or PER, directly linking GSH synthesis and the circadian clock (19).

NRF2 (nuclear factor erythroid 2-related factor 2) is a transcription factor that drives the transcription of several genes involved in antioxidant protection such as those involved in glutathione synthesis (GCL and GS) and glutathione regeneration (GR) (20). In the rat lung, BMAL1 and CLOCK regulates the transcription of NRF2 which in turn drives the expression of genes involved in GSH synthesis (17). NRF2-deficient mouse embryonic fibroblasts were shown to exhibit loss of GCL mRNA rhythms, which in turn corresponded to reduced GSH levels (17). Moreover, in the lungs of mutant mice in which CLOCK expression is disrupted *(Clock^Δ19^ mice*), the rhythmic expression of NRF2 protein was lost along with reduced GCL mRNA expression and lower levels of GSH resulting in increased oxidative damage (17), demonstrating a link between the circadian clock, the NRF2/GSH pathways and protection from oxidative stress.

There is evidence that the lens utilises circadian rhythms to regulate important functions such as the synthesis of the antioxidant melatonin (21–23). In rat lenses, activity of melatonin synthesis enzymes and melatonin levels were highest at night and lowest during the day (21). Moreover, in more recent studies, Chhunchha et al. revealed that the rhythmic expression of NRF2 and one of its target genes, peroxiredoxin 6 (PRX6) was disrupted when BMAL1 expression was knocked down in human lens epithelial cells, resulting in increased ROS levels (24). These results demonstrate that BMAL1 is important for the regulation of NRF2-mediated antioxidant protection. Since NRF2 is also known to regulate expression of genes involved in the synthesis and regeneration of GSH, in this study, we examined whether the BMAL1/NRF2 pathway could play a role in the regulation of GSH levels in the lens and help anticipate the need for higher levels of GSH protection at different times of the day.

## 2 Methods

### 2.1 Reagents

Phosphate-buffered saline (PBS) was prepared from PBS tablets (Sigma-Aldrich Corp., St. Louis, MO, USA). Primers for Bmal1, Clock, Per 1-3, Cry1-2 and β-actin were synthesised by Integrated DNA Technologies (IDT™, Iowa, USA). Primers were reconstituted in RNase/DNase distilled water to yield a 100µM stock solution, which was diluted to 20µM, for use in PCR reactions. BMAL1, PER1, GCLC and β-actin primary antibodies were purchased from Abcam (Cambridge, UK), CLOCK and GR were purchased from ThermoFisher Scientific (Waltham, Massachusetts, USA) and CRY1, CRY2, PER2, PER3, NRF2 and GS were purchased from ProteinTech (Rosemont, Illinois, USA). The goat anti-rabbit Alexa Fluor 488 secondary antibody, the membrane marker wheat germ agglutinin (WGA) conjugated to Alexa Fluor 594 and 4′,6-diamidino-2-phenylindole (DAPI) were obtained from Life Technologies (Carlsbad, CA, USA). Unless otherwise stated, all other chemicals were obtained from Sigma-Aldrich Corp.

### 2.2 Animals

All animals were treated in accordance with protocols approved by the University of Auckland Animal Ethics Committee (Ethics number R001413) and in compliance with the Association for Research in Vision and Ophthalmology (ARVO) Statement for the Use of Animals in Ophthalmic and Vision Research. 6-week-old male Wistar rats were housed in a 12hr/12hr light-dark cycle with the lights turned on at 6am (ZT0) and the lights turned off at 6pm (ZT12), in which ZT refers to Zeitgeber time; a standardised unit of time based on the 12hr/12hr light-dark cycle. Animals were euthanised by CO_2_ asphyxiation and eyes nucleated at either 6am (ZT0), 10am (ZT4), 2pm (ZT8), 6pm (ZT12), 10pm (ZT16) or 2am (ZT20). For the 10am and 2pm collection, enucleation was performed under standard lighting conditions, but for the 6am and 6pm time points, enucleation was performed under dimmed lighting conditions. For the 10pm and 2am time point, a cohort of animals were reverse entrained to enable tissue collection during the day. A least 2 weeks prior to tissue collection, a cohort of animals were maintained in a reverse light-dark cycle – in which lights were turned off at 6am and lights were turned on at 6pm. This period of 2 weeks reverse entrainment was reported to be sufficient to ensure adaptation of animals to a reverse light-dark cycle (25) meaning that we were able to collect tissues at 10am and 2pm instead of 10pm and 2am. In these instances, eyes were enucleated under dim conditions.

### 2.3 Aqueous humour collection

Following enucleation, eyes were transferred immediately to a container prefilled with warm PBS. Eyes were then taken out and placed on a pre-chilled petri dish. A 27- gauge needle was then used to make an initial piercing at the limbus and a 2μL pipette was used to quickly collect the aqueous humour (AH) (roughly 1μL/eye) from both eyes of an animal, pooled together and then placed into a pre-chilled Eppendorf tube (n=6 AH sample for each time point). Tubes were snap frozen immediately in liquid nitrogen and were placed in a -80°C freezer for the quantification of total GSH (GSH + GSSG), reduced GSH and oxidised GSH (GSSG) levels using liquid chromatography tandem mass spectrometry (LC-MS/MS).

### 2.4 Lens dissection

Following enucleation, lenses were dissected from the eye. Lenses collected for RNA extraction were placed in TRIzol Reagent (Invitrogen, Waltham, Massachusetts, USA) and then stored at -80°C. Lenses collected for western blot analysis were homogenised in homogenising solution and then stored at -80°C. Lenses collected for immunohistochemical analysis were fixed in paraformaldehyde. Lenses collected for LC-MS/MS were homogenised in 200μL of 50mM EDTA, spun at 14,000 rpm for 20 minutes at 4°C, supernatants collected, snap frozen and stored immediately in the -80°C freezer.

### 2.5 Real time-polymerase chain reaction

Total RNA was isolated from brain (control tissue), or lenses collected at 10am using Trizol according to the manufacturer’s protocol (TRIzol reagent; Life Technologies). Genomic DNA was removed by incubation with 10U/μL recombinant DNase I (Roche Diagnostics, Basel, Switzerland). Total brain or lens cDNA were synthesised from 1 μg total RNA mixed with 50μM oligo(dT)_20_. The RNA was denatured at 65°C for 5 minutes, immediately placed on ice to cool, and then combined with 2× First-Strand Reaction Mix and SuperScript III/RNaseOUT Enzyme Mix (Life Technologies) for cDNA amplification. A control reaction (no cDNA synthesis) was also conducted in the absence of SuperScript III/RNaseOUT enzyme. Synthesised cDNA or control reaction (0.5–1μL) were added to separate PCR mixtures containing final concentrations of 5μL PowerUpTM SYBR Green PCR Master Mix (Applied BiosystemsTM, Waltham, Massachusetts, USA) and 3μL RNase-free water to a final volume reaction mixture of 10μL and 2 μM sense and antisense primers^15,16^ (**Table 1**). The qPCR reaction was 95°C for 10 minutes, 40 cycles of denaturation at 95°C for 15 seconds, and a combined annealing/extension at 60°C for one minute. The mRNA relative quantity of target genes was obtained from the method of comparative threshold cycle (CT) and the target gene level of expression were normalised to the β-actin levels as an endogenous control within each group.

**Table 1:** Primer sequences for target genes.

Gene Name (GenBank Accession #)	Primer Sequence (5’ – 3’)	Amplicon Size (bp)
**β-actin** (NM_001101.5)	Forward: AGCCATGTACGTAGCCATCC Reverse: TCTCAGCTGTGGTGGTGAAG	171
**Bmal1** (NM_024362)	Forward: CCGATGACGAACTGAAACACCT Reverse: TGCAGTGTCCGAGGAAGATAGC	215
**Clock** (NM_021856)	Forward: TCTCTTCCAAACCAGACGCC Reverse: TGCGGCATACTGGATGGAAT	110
**Cry1** (NM_198750)	Forward: TGCTCCTGGAGAGAATGTCC Reverse: TGACTCTCCCACCAACTTCA	271
**Cry2** (NM_133405)	Forward: GGATAAGCACTTGGAACGGAA Reverse: ACAAGTCCCACAGGCGGT	155
**Per1** (NM_001034125)	Forward: ACACCCAGAAGGAAGAGCAA Reverse: GCGAGAACGCTTTGCTTTAG	164
**Per2** (NM_031678)	Forward: GAGAGAGGAACAGGGCTTCC Reverse: TTGACACGCTTGGACTTCAG	195
**Per3** (NM_023978)	Forward: ATAGAACGGACGCCAGAGTGT Reverse: CGCTCCATGCTGTGAAGTTT	104
**Nrf2** (NM_031789)	Forward: GTTGAGAGCTCAGTCTTCAC Reverse: CAGAGAGCTATCGAGTGACT	56
**Gclc** Catalytic Subunit (NM_012815)	Forward: ATCTGGATGATGCCAACGAGTC Reverse: CCTCCATTGGTCGGAACTCTACT	129
**Gs** (NM_012962.1)	Forward: GCAGGAACTGAGCAGGGTG Reverse: GCTTCAGCACAAAGTGGCTAG	169
**Gr** (NM_053906.2)	Forward: GGGCAAAGAAGATTCCAGGTT Reverse: GGACGGCTTCATCTTCAGTGA	101

### 2.6 Western blotting

Lenses (n=16 lenses) and positive control tissue (kidney, liver or brain; n=1) were collected at 10am and homogenised in homogenising solution (5mM Tris-HCl, 5mM EDTA, and 5mM EGTA (pH 8.0) containing cOmplete Protease Inhibitors (Roche, Basel, Switzerland). Homogenates were centrifuged at 13,000g for 20 minutes and the supernatant stored at −80°C until further use. Concentrations of proteins were determined using the Direct Detect Infrared Spectrometer (Merck, Millipore). Proteins (20µg/lane) were first separated on a 10% or 15% vol/vol acrylamide separating gel and then transferred onto the Immuno-Blot PVDF membrane (Bio-Rad Laboratories) by electrophoresis. After transfer, membranes were incubated with blocking solution (5% milk powder in 1× Tris-buffered saline with Tween 20, pH7.6) for 1 hour and then incubated with primary antibodies (BMAL1 (1:500), CLOCK (1:200), CRY1 (1:1000), CRY2 (1:500), PER 1 (1: 500), PER 2 (1:500), PER 3 (1: 500), NRF2 (1:500), GCLC (1:500), GS (1:1000) or GR (1:500) overnight at 4°C. Membranes were incubated with donkey anti-rabbit secondary antibodies (1:10,000) for 1 hour. Labelled protein was visualised using enhanced chemiluminescence detection (ECL Prime; GE Healthcare) and developed using the Fujifilm Luminescent Image Analyser LAS-4000 System (GE Healthcare).

To determine if protein expression changed over a 24-hour period, lenses were collected at 6am (ZT0), 10am (ZT4), 2pm (ZT8), 6pm (ZT12), 10pm (ZT16), 2am (ZT20) (n=16 lenses for each time point) and processed as described above. Proteins were electrophoresed and transferred to membranes where the following primary antibodies were used: BMAL1 (1:500), CLOCK (1:200), CRY2 (1:500), NRF2 (1:500), GCLC (1:500), GS (1: 1000) or GR (1:500). Membranes were then incubated with secondary antibodies and labelled protein visualised as described above. Equal protein loading was tested by stripping the membranes with 2% SDS, 100 mM β-mercaptoethanol, 62.5mM Tris (pH 6.7) and then re-probing the membrane with antibodies to detect β-actin (1:1000).

### 2.7 Immunohistochemistry

Whole lenses collected at 10am (ZT4), 2pm (ZT8), 6pm (ZT12) and 10pm (ZT16) (n= 4 lenses for each time point) were fixed in 0.75% wt/vol paraformaldehyde, cryoprotected, and cryosectioned in an axial orientation using standard protocols developed in our laboratory. Sections were washed three times and incubated in blocking solution (3% wt/vol bovine serum albumin and 3% vol/vol normal goat/donkey serum) for 1 hour to reduce nonspecific labelling. The sections were then labelled with either BMAL1 (1:200), CLOCK (1:200) or NRF2 (1:100) antibodies diluted in blocking solution, followed by the goat anti-rabbit Alexa Fluor 488 (1:200) secondary antibody for 2 hours. To highlight cell morphology, cell membranes were labelled with WGA Alexa Fluor 594 (1:100) in PBS and to highlight epithelial and fiber cell nuclei, sections were stained with DAPI (1:10,000). Sections were then washed and mounted with VECTASHIELD HardSet aqueous mountant (Vector Laboratories, Burlingame, CA, USA) and viewed using an Olympus FV1000 confocal laser scanning microscope (Olympus Corporation, Tokyo, Japan). To facilitate comparison between data sets, the same pinhole size was used. Specific emission filter sets were used to detect signals from Alexa Fluor 488, WGA Alexa Fluor 594, and DAPI fluorophores.

### 2.8 Quantification of GSH levels in aqueous humour and lenses using LC-MS/MS

LC-MS/MS was used to quantify GSH and GSSG in the AH and lens as previously described (26). Samples were collected at six different time points (6am (ZT0), 10am (ZT4), 2pm (ZT8), 6pm (ZT12), 10pm (ZT16) and 2am (ZT20)) over a 24-hour period. Briefly, lens supernatants and AH samples were first thawed on ice. Known concentrations (calibration curve) of GSH and GSSG, internal standards (isotopically labelled GSH (13C 15 N)) and GSSG (13C 15 N) as an internal quality control), and AH and lens samples were immediately treated with monobromobimane (MBrB). Samples were then added to a previously conditioned solid phase extraction cartridge (Strata-X-C, Phenomenex, Torrance, CA, USA) before being eluted in 5% NH4OH. Known and unknown samples were vacuum-concentrated and reconstituted in 5% acetonitrile/0.1% heptafluorobutyric anhydride in H_2_O. Separations were then performed by injecting 10µL sample into the LC equipped with a Phenomenex Synergi Hydro-RP C18 4μm 150 × 2mm column (Phenomenex, Torrance, CA, USA) and a 0.2μm in-line filter (Phenomenex, Torrance, CA, USA) in gradient mode. The column effluent was then directed into an Agilent 6460 A Triple Quadrupole mass spectrometer (Agilent Technologies, Santa Clara, California, USA) with parameters set in **Table 2**. GSH and GSSG were quantified using the calibration curve with known concentrations of CSH (range 0–100μM), CSSC (range 0–50μM), GSH (range 0–400μM) and GSSG (range 0–50μM). Metabolite concentrations were expressed as μM and normalised to lens wet weight as individual fractions, particularly of the epithelium, were too difficult to accurately measure.

**Table 2:** MS/MS ions and parameters.

Analyte	Mass transition	Fragmentation Voltage	Collision Energy
GSH-mBrB	498.2>435.1	185 V	21 V
	498.2>192.1	185V	45 V
GSH-mBrB (13C 15N)	501.2>438.1	185 V	21 V
	501.2>192.1	185 V	45 V
GSSG	613>355	190 V	22 V
	613>484	190 V	15 V
GSSG (13C 15N)	619>361	190 V	22 V
	619>490	190 V	15 V

### 2.9 Statistical analysis

All numerical values and graphs are displayed as mean ± standard error of the mean (SEM) unless otherwise stated. To compare gene expression between the brain and lens, for each target gene, a t-test was conducted to determine statistical significance. To compare changes across the 24 hour period, a one-way ANOVA test was first conducted to determine statistical significance. Once significance was confirmed, a Tukey’s *post-hoc* analysis was conducted to determine significance between groups using GraphPad Prism^®^ Version 8. P values of <0.05 were considered statistically significant. To compare differences between GSH levels in the lens and AH at different time points, GSH concentration in the lens and AH was rescaled against the max concentration in either the lens or AH and tabulated. A two-way ANOVA along with the Bonferroni multiple comparison test was then conducted to determine significance at each time point. P values of *p<0.05, **p<0.01 or ***p<0.001 were considered statistically significant.

## 3 Results

### 3.1 Identification of clock component genes in the rat lens

**Figure 1:**
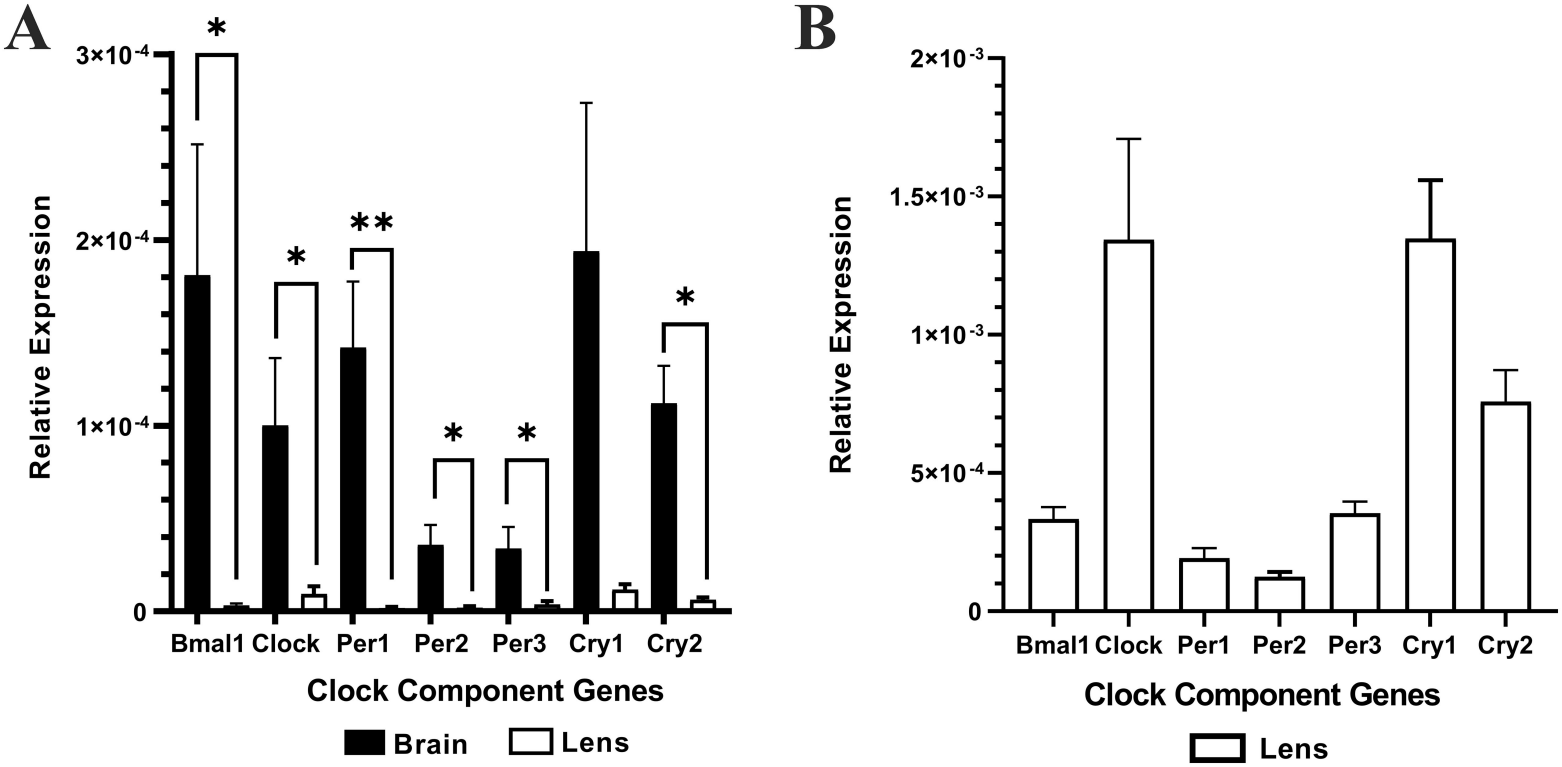
The relative expression of the clock component genes in the brain and lens. Brain and lens tissue were harvested at 10am and RNA extracted for cDNA synthesis. RT-qPCR in combination with isoform specific primers for the clock component genes was then performed. **(A)** The relative expression of clock component genes in the brain compared to the lens. **(B)**. Rescaled version of **(A)** to show the relative expression of clock component genes in the lens only. The relative expression for each clock component gene was calculated using the DCt method where the mean Ct value of the target gene is normalised to the b-actin internal control. Data is presented as mean relative expression ± the standard error of the mean (± SEM) from n = 4 rats. *p<0.05; **p<0.01.

Brain (positive control) and lens tissue was collected from the same animal at the same time (10am (ZT4)) and total RNA extracted. Brain or lens cDNA was synthesised and then used as a template for RT-qPCR using isoform specific primers for the clock component genes; Bmal1, Clock, Per1, Per2, Per3, Cry1, and Cry2. The relative expression of each clock component gene was normalised to the internal control β-actin. Lens -RT reactions where reverse transcriptase was omitted produced no amplification plots and therefore no relative expression values were provided. **Figure 1A** shows the relative expression of clock component genes in the brain compared to the lens (n =4 rats). While the lens expresses all the clock component genes, the levels of expression are significantly lower compared to that of the brain. A closer look at the expression levels in the lens (**Figure 1B**) revealed some interesting findings compared to the brain. Firstly, for Bmal1 and Clock in the lens, Clock expression appeared higher compared to Bmal1 expression. This was different to the pattern in the brain in which Bmal1 was more highly expressed compared to Clock. However, these differences were not statistically significant. Secondly, in the lens, all three Per isoforms appear to be expressed at similar levels, whereas in the brain Per1 appeared to be the more abundant isoform. Finally, like the brain, Cry1 expression appeared to be slightly higher relative to Cry2 expression in the lens. However, these differences were not statistically significant. Taken together, this is the first time that expression of all these clock component genes have been identified in the rat lens.

### 3.2 Expression of core clock component proteins in the rat lens

**Figure 2:**
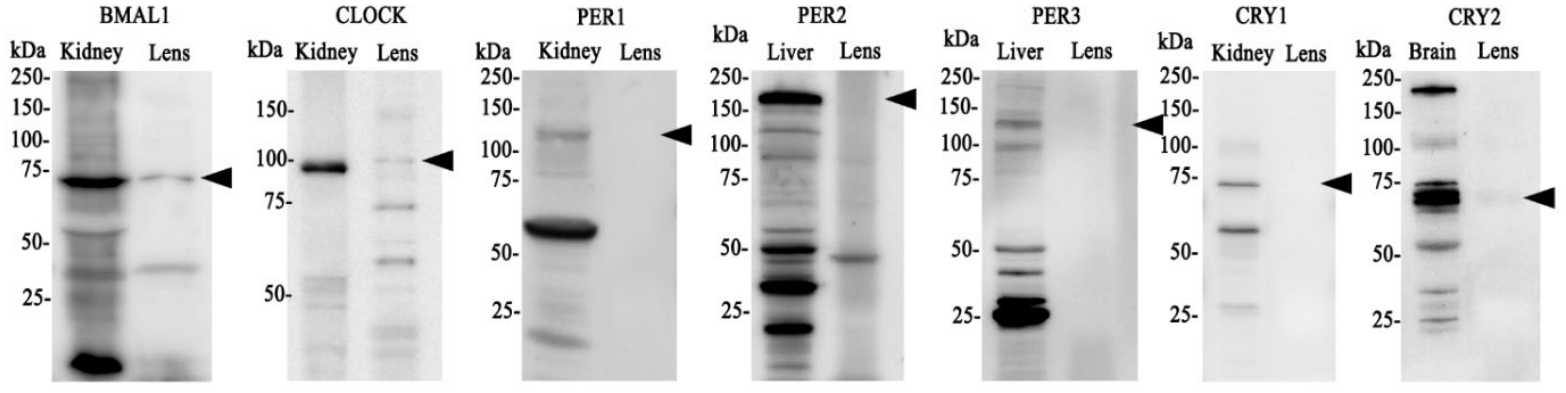
Protein expression of clock components of the positive and negative arm of the circadian clock in the rat lens. 20µg/lane of positive control tissue (kidney, liver or brain (n= 1 rat)) and 20µg/lane lens tissue (n=8 rats) was electrophoresed on an SDS-PAGE gel and protein levels analysed by Western blotting. Expression of BMAL1, CLOCK, PER1, PER2, PER3, CRY1 & CRY 2. Arrowheads indicate the predicted size of the target protein. Target proteins were all identified in positive control tissue but not all could be detected in the lens.

Having established that clock component genes are expressed in the lens, we next investigated whether BMAL1, CLOCK, PER1-3 and CRY1-2 are expressed at the protein level (**Figure 2**). Brain, kidney or liver (positive control) and lens tissue was collected at the same time (10am (ZT4)) and protein extracted. We originally used brain tissue as control but found it hard in some cases to identify bands of interest, so opted to test two different control tissues: kidney and liver. Because clock cycling can differ in peripheral tissues versus the brain it was more likely we would detect all of our clock proteins if we used more than one control tissue. As was the case, we were able to detect all core clock proteins in control tissue (kidney, liver or brain). However, in the lens, we only detected bands of the appropriate molecular weight for BMAL1 and CLOCK (**Figure 2**). While we loaded an equal amount of protein in the kidney and lens, the band for BMAL1 and CLOCK was more intense in the kidney relative to the lens, suggesting these clock proteins are more abundantly expressed in the kidney. While we detected a faint band for CRY2, we were unable to detect bands for CRY1 or PER1-3. This might suggest that time of day differences exist in the expression of these clock proteins in the lens, with BMAL1 and CLOCK expression more abundant during the day (10am) relative to CRY and PER expression.

### 3.3 Expression and localisation of core clock component proteins in the lens at different times of the day

**Figure 3:**
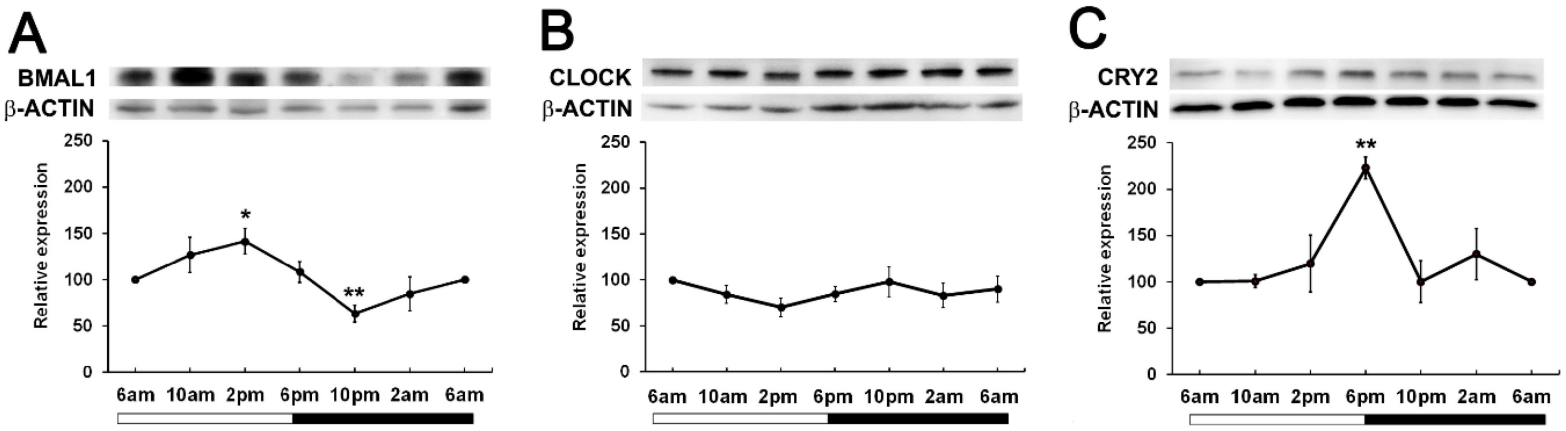
Expression of clock component proteins in the rat lens at different times of the day. **(A)** Expression of BMAL1, **(B)** CLOCK and **(C)** CRY2 over a 24-hour time period. Lens tissue (n=8 rats) was harvested at 4-hour intervals over a 24-hour period. 20µg lens protein/lane was electrophoresed on an SDS PAGE gel and protein levels analysed by Western blotting and expressed relative to the β-actin internal control. Relative expression of BMAL1, CLOCK and CRY2 expression in lenses at different times. Data is presented as mean relative expression ± standard error of the mean (SEM) from 5-7 western blots. * Indicates significant differences from the 6am time point; *p<0.01; **p<0.005.

To determine if time of day differences existed in clock protein expression, we investigated whether the expression of BMAL1, CLOCK and CRY2 oscillated over a 24-hour period. Wistar rats were housed in a 12hr/12hr light-dark cycle with the lights turned on at 6am and the lights turned off at 6pm. Lenses were collected at 4-hour intervals starting at 6am (ZT0) over a 24-hour period and then clock protein expression at 6am (ZT0), 10am (ZT4), 2pm (ZT8), 6pm (ZT12), 10pm (ZT16) and 2am (ZT20) examined by western blotting. BMAL1 expression changed over the course of the 24-hour period, with expression significantly increased at 2pm relative to the 6am time point. BMAL1 expression then declined to reach a significant trough at 10pm relative to the 6am time point (**Figure 3A**). On the other hand, CLOCK expression remained unchanged over the 24-hour period (**Figure 3B**). CRY2 expression was also seen to change over the course of the day with expression levels peaking at 6pm which was significantly increased relative to the 6am time point (**Figure 3C**). Since BMAL1 expression was higher during the day and CRY2 expression lower during the day, this may explain why it was difficult to initially detect CRY2 at 10am (**Figure 2**).

### 3.4 Expression of NRF2 and NRF2-regulated GSH related proteins involved in GSH homeostasis

**Figure 4:**
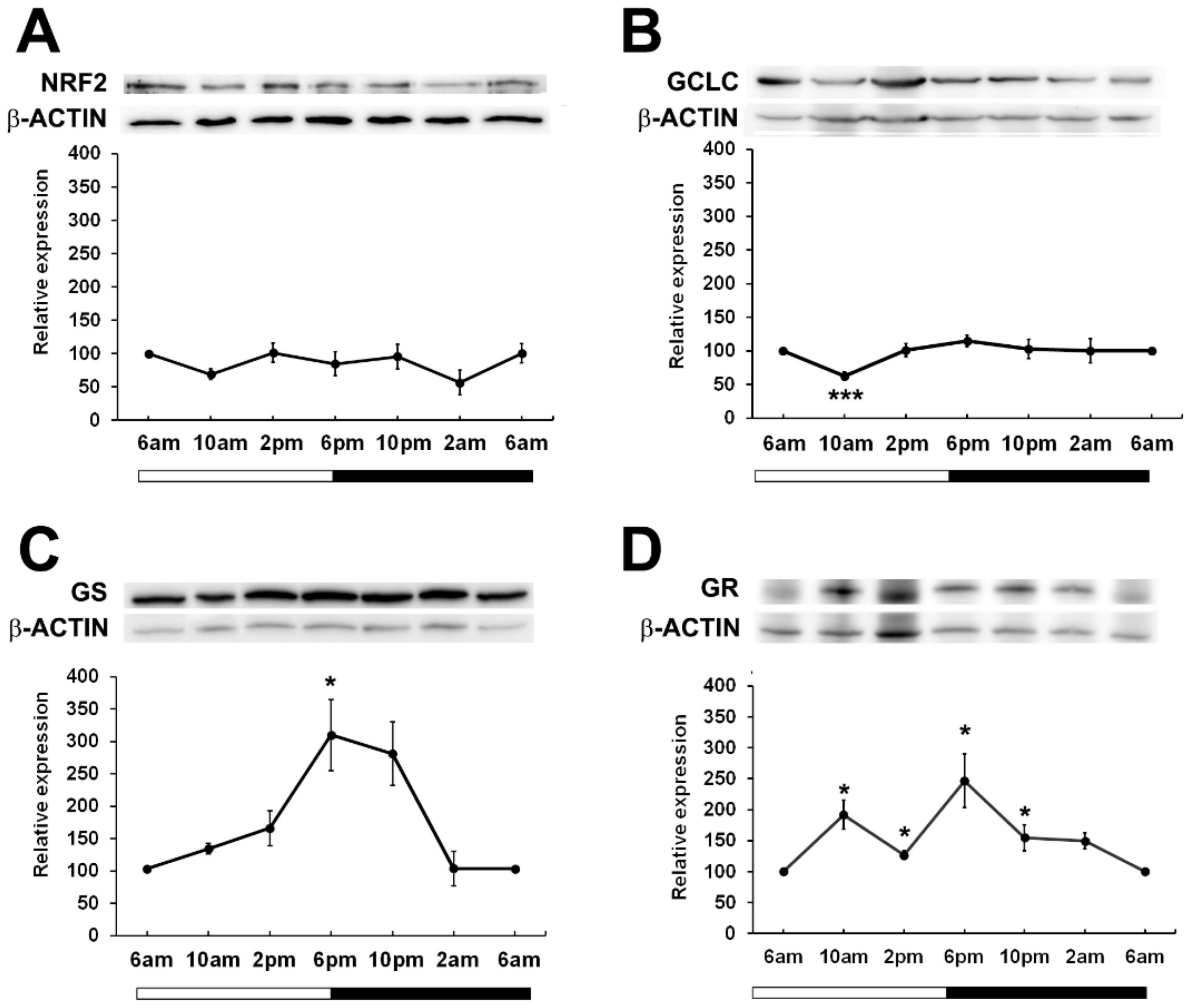
Relative expression of NRF2 and NRF2 related proteins in the rat lens over a 24-hour period. Lens tissue (n=8 rats) was harvested at 4-hour intervals over a 24-hour period. 20µg lens protein/lane was electrophoresed on an SDS-PAGE gel and protein levels were analysed by Western blotting and expressed relative to the β-actin internal control. Relative expression of **(A)** NRF2, **(B)** GCLC, **(C)** GS and **(D)** GR in lenses at different time points. Data is presented as mean relative expression ± standard error of the mean (SEM) from 5-7 western blots. * Indicates significant differences from the 6am time point; *p<0.05, ***p<0.001.

Given that in other tissues, BMAL1 is important in the regulation of NRF2 (17) which drives the transcription of genes involved in the synthesis and regeneration of GSH, we examined the expression patterns of NRF2 over a 24-hour period. While it was expected that NRF2 expression may peak and trough in phase with BMAL1 as had been previously reported in mouse lenses (24), we found that in rat lenses, NRF2 expression remained constant over the 24-hour period (**Figure 4A**). To determine whether GSH related proteins linked to NFR2 regulated transcription showed time of day differences in their expression patterns, we examined the expression of Glutamate-Cysteine Ligase (GCLC) -the enzyme involved in the first step of GSH synthesis, Glutathione Synthetase (GS) - the enzyme involved in the second step of GSH synthesis, and Glutathione Reductase (GR) -the enzyme involved in the regeneration of GSH (**Figures 4B–D**). GCLC expression significantly decreased at 10am relative to 6am, and then slowly increased at 2pm and remained constant over the 24-hour period (**Figure 4B**). On the other hand, GS expression increased from 6am through to 6pm, with expression significantly increased at the start of night (6pm) relative to the 6am time point. (**Figure 4C**). GR levels fluctuated over the 24-hour period, with expression significantly increased at 10am and at 6pm relative to the 6am time point (**Figure 4D**). Taken together, it appears that GSH related enzyme expression generally increased at the start of the dark period which would coincide with the start of the active phase of the nocturnal rat.

### 3.5 Time of day differences in the subcellular localisation of BMAL1, CLOCK and NRF2

**Figure 5:**
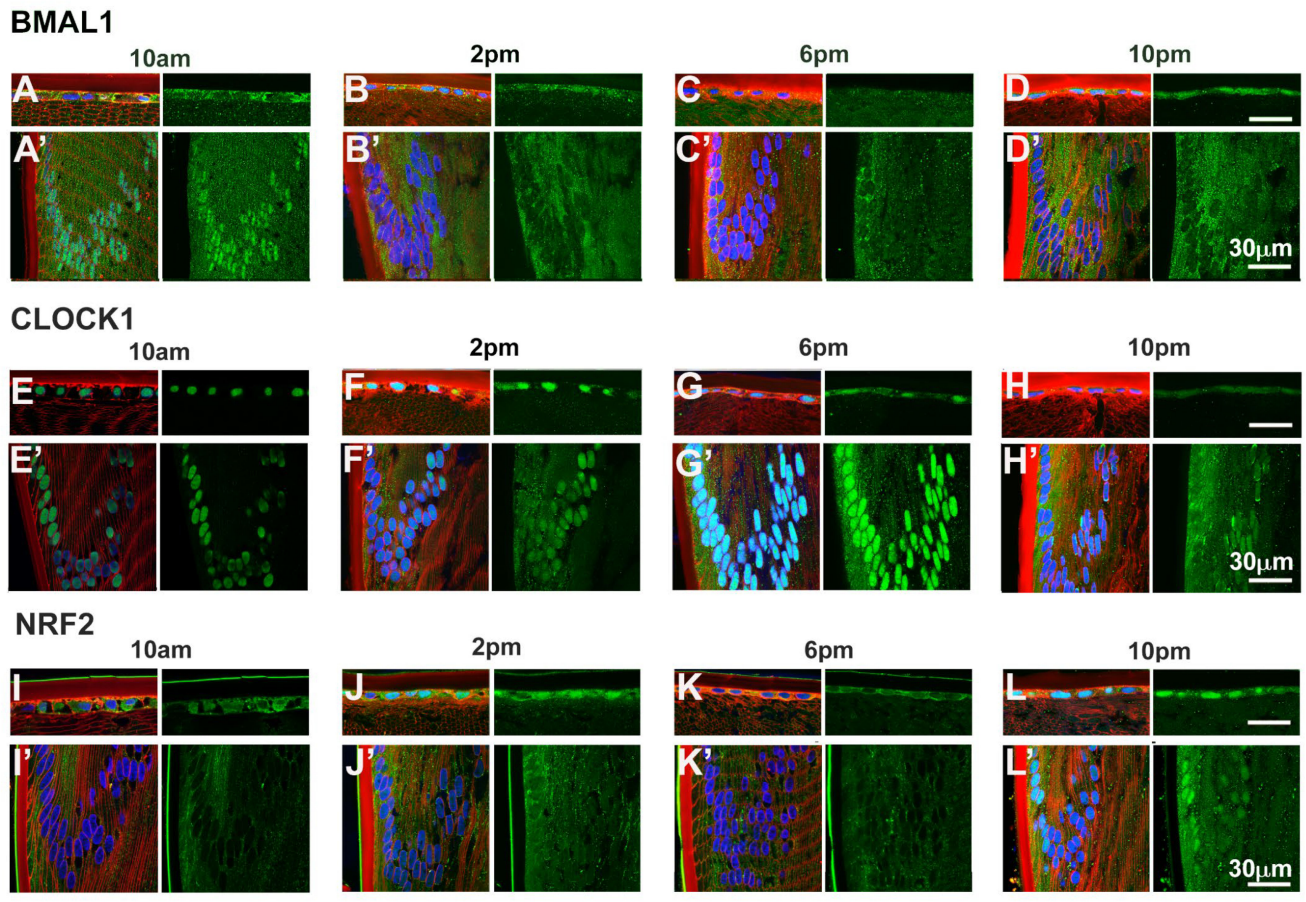
Expression of clock component proteins and NRF2 in the rat lens at different times of the day. Subcellular localisation of BMAL1, CLOCK and NRF2 at different times of the day. Lenses were dissected at 10am, 2pm, 6pm or 10pm, fixed, cryoprotected and cryosectioned in an axial orientation. Images were taken at either the anterior pole **(A–L)** or the lens equator **(A’-L’)**. **(A–L)** *Left hand panels*-sections labelled with the membrane marker WGA (*red*), DAPI (*blue*) and BMAL1, CLOCK or NRF2 antibodies (*green*). **A’-L’**: *Right hand panels* -sections showing only BMAL1, CLOCK or NRF2 labelling. *n* = 4 lenses.

Given that BMAL1, CLOCK and NRF2 are transcription factors, we investigated the subcellular localisation of these proteins as localisation to the nuclei might suggest active transcription of genes at a particular time of the day. To do this, we labelled axial sections from lenses collected at four different time of the days; 10am (ZT4), 2pm (ZT8), 6pm (ZT12) and 10pm (ZT16) with antibodies specific for BMAL1, CLOCK or NRF2 (*green*), DAPI (*blue*) to visualise the nuclei, and WGA (*red*) to highlight the membranes of the epithelial and fiber cells. These sections were then visualised under a confocal microscope and images taken at the anterior pole (**Figures 5A–L**) and at the equator region (**Figures 5A’–L’**). At the anterior pole, BMAL1 labelling was mainly detected in the epithelium, with less labelling evident in the fiber cells for each time point (**Figures 5A–D**). While BMAL1 labelling was mainly cytoplasmic, at 2pm and 10pm, BMAL1 could be seen to be co-localised to the nuclei (**Figures 5B, D**). At the lens equator, BMAL1 labelling was detected in the epithelial and fibre cells for each time point (**Figures 5A’–D’**). While BMAL1 was predominantly localised to the cytoplasm for each time point, at 10am BMAL1 was strongly co-localised to the nuclei (**Figure 5A’**). At the anterior pole and equator region, CLOCK was strongly associated with the nuclei for each time point (**Figures 5E–H, E’–H’**). Like CLOCK, NRF2 was co-localised to the nuclei of epithelial cells at the anterior pole for each time point (**Figures 5I–L**), but at the lens equator, NRF2 appeared to translocate in and out of the nuclei at the different timepoints (**Figures 5I’–L’**). At 10am, NRF2 was absent from the nuclei (**Figure 5I’**), which was different to what was observed for BMAL1 and CLOCK at this same time point (**Figures 5A’, E’**). However, at 2pm, NRF2 was co-localised to the nuclei, then was absent from the nuclei at 6pm and then reappeared in the nuclei at 10pm (**Figures 5J’–L’**).

### 3.6 GSH levels fluctuate at different times of the day in the lens and aqueous humour

**Figure 6:**
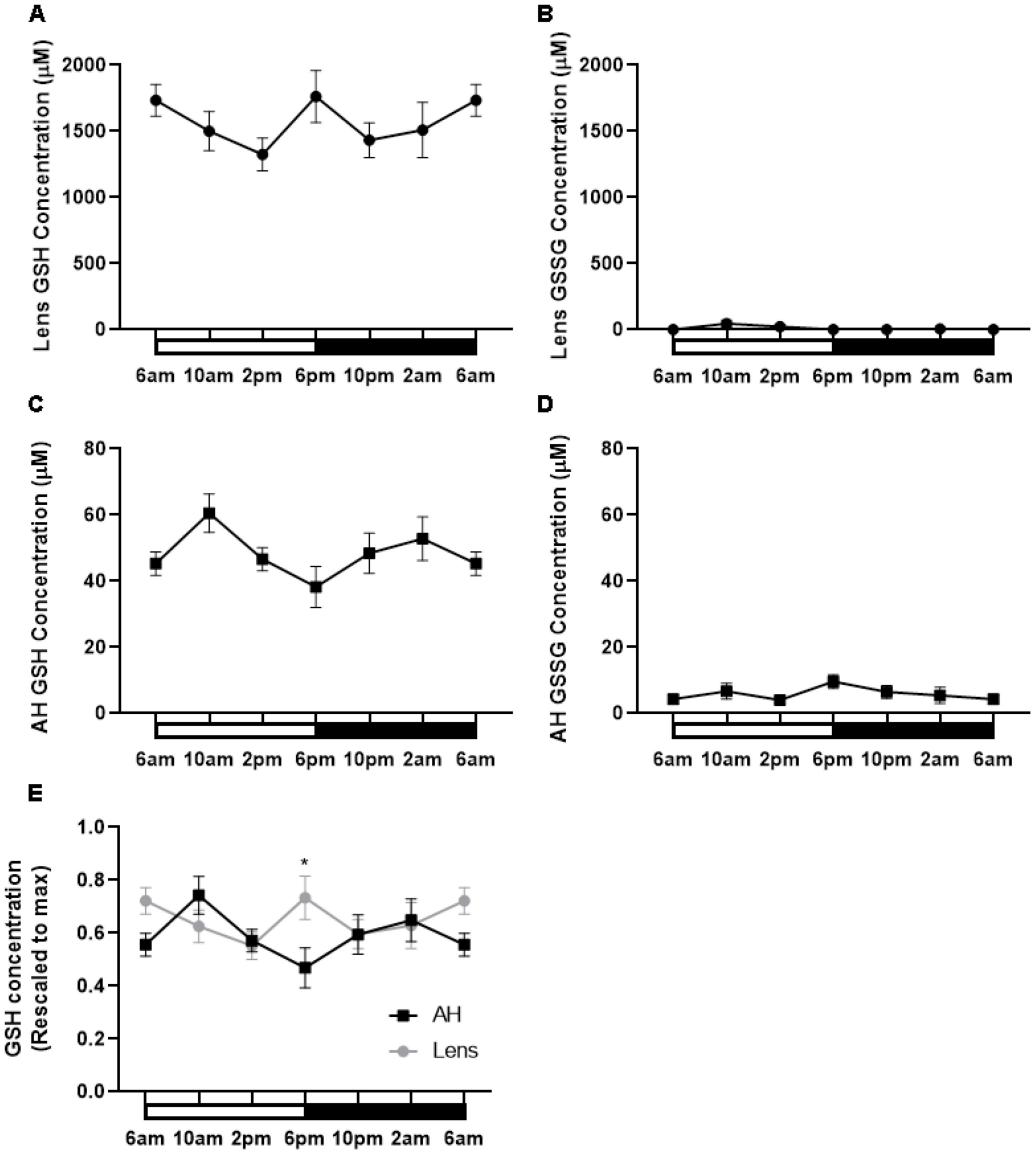
GSH levels in the rat lens and aqueous humour over a 24- hour period. **(A, B)** Lenses and **(C, D)** aqueous humour (AH) were collected from six-week-old male Wistar rats at six different time points (6am, 10am, 2pm, 6pm, 10pm and 2am) over a 24-hour period and analysed by LC-MS/MS to quantify the concentration of GSH **(A, C)** and GSSG **(B, D)**. **(E)** Concentration of GSH in the AH and Lens rescaled to max concentration. Each point and error bars represent the mean ± S.E.M. (n=6 animals). *p<0.05.

Having shown that the expression of enzymes involved in GSH synthesis appeared to increase at night, we determined if this corresponded to higher levels of GSH during the dark period. To investigate this, lenses were collected at six different time points (6am (ZT0), 10am (ZT4), 2pm (ZT8), 6pm (ZT12), 10pm (ZT16), 2am (ZT20)) from male Wistar rats over a 24-hour period and GSH/GSSG concentrations measured via LC-MS/MS (**Figure 6**). In the lens, the majority of GSH was in the reduced form relative to the oxidised form. Reduced GSH levels in the lens appeared high at 6am and to then decrease at 2pm, before increasing at 6pm which correlates to the start of night. From here, GSH levels decrease at 10pm and then levels remain steady overnight (**Figure 6A**). While an obvious trend was seen, this was not statistically significant. GSSG levels in the lens were almost negligible with no time-of-day differences seen (**Figure 6B**). While the lens can synthesise GSH, it is known that the lens can take up GSH directly from the aqueous humour (AH). To investigate time-of-day differences in GSH levels in the AH, we collected AH at six different time points (6am, 10am, 2pm, 6pm, 10pm, 2am) over a 24-hour period and GSH/GSSG concentrations measured. Like the lens, the majority of GSH in the AH was in the reduced form relative to the oxidised form. While not statistically significant, there was a trend with low levels of GSH detected at 6am before increasing to a peak at 10am, decreasing from 10am to 6pm, before gradually increasing and then plateauing at 6am (**Figure 6C**). GSSG levels were low compared to GSH levels and did not appear to change over time (**Figure 6D**). At 6pm where GSH levels in the AH are lowest (**Figure 6C**), GSH levels in the lens appear highest (**Figure 6A**). To compare these differences, individual GSH concentrations for each time point were rescaled against the max value in either the lens or the AH and plotted (**Figure 6E**). At 6pm, the levels of GSH in the lens were significantly different to the levels of GSH in the AH (p=0.03). A similar trend was seen at 6am where high levels in GSH in the lens appeared to correspond to low levels of GSH in the AH. However, this was not statistically significant.

## 4 Discussion

Traditionally there has been a strong bias in basic research on circadian rhythms towards the use of male animals in studies with less than 20% of work in this area including female cohorts (27). To determine in the first instance if the rat lens contained the machinery of a circadian clock, we opted to use a use male rats, so that we could compare our findings to the existing literature. In this study, we confirmed the presence of clock genes from the positive (Bmal1 and Clock) and negative arms (Per1-3, Cry1-2) of the circadian clock in the rat lens (**Figure 1**). The expression of these core clock genes was significantly lower than that of the brain (**Figure 1A**). However, it is worth noting that since most cells are anucleate, a homogenate of whole lenses might appear to show less clock gene expression than brain because only a subset of lens cells express those clock genes whereas all brain cells express clock genes. Therefore, it might still be possible that those individual lens cells that are expressing clock components are expressing them to a same level as individual brain cells. Another possibility is that lens and brain clocks may not necessarily be in sync. Nevertheless, the identification of Bmal1 and Clock in the rat lens supports another study that identified Bmal1 and Clock at the mRNA level in human lens epithelial cells and mouse lenses (24). However, to our knowledge, this is the first report of components of the negative arm of the circadian clock being identified in the lens. The detection of all Per and Cry isoforms in the lens is consistent with other studies where it is common for Per 1-3 and Cry 1-2 to be expressed in the same tissue; for example, Per1-3 mRNA are all expressed in the SCN (28, 29), lungs (30), liver (31), and cartilage (32), and Cry 1 and 2 mRNA are expressed in tissues such as the SCN (33), lung (34) and liver (35). Taken together, the detection of both the positive and negative limb of the circadian transcriptional-translational feedback loop supports the idea that the lens may contain its own circadian clock.

At the protein level, BMAL1, CLOCK and CRY2 were detected in the lens, but CRY1 and PER1-3 was undetectable (**Figure 2**), suggesting that these proteins may be more abundantly expressed during the night compared to during the day. Examining the expression of clock proteins at 4-hour intervals over a 24-hour period and their subcellular localisation during the light (10am) and the start of the dark period (6pm), revealed that BMAL1 expression was highest during the light period (6am-2pm) (**Figure 3A**), which corresponded with the nuclear localisation of BMAL1 at 10am (**Figure 5A’**). This is consistent with studies in other peripheral tissue cells, where BMAL1 levels and/or activity have been shown to be highest during the day and lowest at night (36–38). In this study, the pattern of expression for BMAL1 in the lens suggests during the light period, BMAL1 may be actively transcribing genes such as Nrf2 and those related to GSH homeostasis.

In contrast to BMAL1 expression in the lens, CLOCK expression did not fluctuate over the 24-hour period (**Figure 3B**) and the subcellular localisation of CLOCK remained nuclear during the light vs dark period (**Figures 5E–H, E’–H’**). This is consistent with the finding in rodent SCN that CLOCK shows stable levels during the 24-hour cycle and is constitutively expressed in the mouse SCN (36, 39). In other tissues, it has been reported that constitutively expressed CLOCK has the potential to make temporally specific associations, alternating between BMAL1 and PER/CRY, thus resulting in transcriptional activation or repression, respectively. In addition, it has also been reported that CLOCK is available to bind to other target proteins such as the p65 subunit of NF-kB (40) which in the lens has been shown to be associated with oxidative-induced damage in human lens epithelial cells (41).

At the protein level, circadian oscillation of clockwork negative factors such as PERs and CRYs were expected to be in anti-phase with BMAL1, and to be more highly expressed during the dark period relative to the night based on studies on mouse SCN (36). In support of this, western blotting of lens samples collected during the day revealed negligible labelling for PER1-3 and CRY 1-2 compared to BMAL1 and CLOCK (**Figure 2**). When examining CRY2 expression over a 24-hour period, it was revealed that expression was relatively low at all time points except at 6pm (**Figure 3C**), confirming that at least for CRY2, its peak expression at night was out of phase with peak BMAL1 expression during the day. Unfortunately, we were unable to replicate this with CRY1 or the PER isoforms. Despite testing with different commercial antibodies, our western blot results were inconsistent, and we could not obtain a reliable pattern of expression (see **Supplementary Figure 1**).

Having established the expression pattern for the core clock protein BMAL1 in the lens, we next examined the expression of NRF2 and GSH related proteins (GCLC, GS and GR) at different times of the day (**Figure 4**). We expected that if BMAL1 was driving NRF2 expression that BMAL1 and NRF2 expression would be in phase with each other as reported in female mouse lenses (24). However, in our study, using male mouse lenses, NRF2 expression remained relatively steady over the 24-hour period (**Figure 4A**). The difference may be due to sexual dimorphism in the expression of Nrf2 (13) where it was reported that Nrf2 transcript expression in the liver was highest during the day than at night in female but not males, with Nrf2 transcript levels also higher in females than in males. In the same manner, NRF2 protein expression might also exhibit sexual dimorphism and explain the lack of obvious time of day differences in NRF2 expression in male lenses. Given that is has become increasingly apparent that sex differences exist in terms of antioxidant defence and the regulation of redox homeostasis (42), it is clear that further studies comparing male and female expression of Nrf2 in the lens should be conducted as if there are sex differences in Nrf2 expression, this might impact GSH regulation.

NRF2 is a transcription factor which induces the transcription of various genes involved in redox balance in response to oxidative stress (43–45). These include GCLC which is involved in the first step of GSH synthesis, GS which is involved in the second step of GSH synthesis and GR that is involved in the regeneration of GSH. Given that we did not see any obvious time of time day differences in the expression of NRF2, it was uncertain whether we would observe differences in NRF2 regulated GCLC and GR expression. However, our studies showed that GCLC expression increased during the later part of the day and through to the dark period (2pm-6am) (**Figure 4B**), while GS and GR levels peaked at the start of the dark period (**Figures 4C, D**). While we did not measure enzyme activity *per se*, these findings indicate that GSH synthesis and regeneration may be higher during the dark period.

Given that BMAL1/CLOCK has been shown to drive NRF2 expression in other tissues (17, 24), we reasoned that examining their subcellular localisation might give us an idea of what time of day these proteins were transcribing genes (**Figure 5**), and whether this correlated to specific regions in the lens which contain nucleated cells: the anterior epithelium which is in the direct pathway of light, and the lens equator, which is not in the direct pathway of light as it covered by the iris, but represents nucleated epithelial and fiber cells area that provide the majority of lens GSH via GSH synthesis (46). However, it was difficult to make a correlation. At the anterior pole and lens equator, CLOCK localised to the nuclei at each time point, while BMAL1 and NRF2 appeared to shuttle in and out of the nuclei at different times. While this might suggest a temporal association between BMAL1 driven transcription/translation of NRF2, we cannot be certain since BMAL1 and CLOCK can each separately bind to other proteins (40, 47) and NRF2 can transcribe genes involved in xenobiotic disposition, protection from electrophiles and general stress response (48, 49). As such, the presence of BMAL1 in the nuclei is not solely indicative of Nrf2 transcription nor the presence of NRF2 in the nuclei solely indicative of transcription of genes involved in GSH homeostasis. However, the finding that GSH levels oscillated over a 24-hour period does provide supportive evidence that regulation of GSH levels may be circadian driven. GSH levels were shown to rise towards the start of night (6pm) which correlates with the higher expression of GS and GR during the dark period. This finding suggests that higher levels of GSH may be required at the start of the active phase of the nocturnal rat. While exogenous ROS sources, such as UV light from sunlight may contribute to the oxidative milieu, endogenous ROS sources such as the mitochondria (50) are likely to be more significant ROS contributors to the nocturnal rat. Therefore, higher levels of GSH can act to directly scavenge ROS or act as a cofactor for antioxidant enzymes such as glutathione peroxidase (GPx), which uses GSH as a cofactor to detoxify hydrogen peroxide (H_2_0_2_) (51).

Measurements of GSH levels in the aqueous humour over a 24-hour period also revealed that GSH levels fluctuate over the course of the day/night. However, the peaks and troughs of GSH in the lens were opposite to that seen in the aqueous humour. GSH levels in the aqueous humour were lowest at the start of the night (6pm) and GSH levels in the lens highest at the start of night. To our knowledge, no other studies have measured GSH levels in the lens and aqueous humour at different times of the day. From our data it is not possible to determine whether GSH levels follow a circadian rhythm per se as since levels peaked at 6am and 6pm this could also indicate the presence of an ultradian rhythm of 12 hours. However, studies have measured intraocular pressure (IOP) where it has been shown that IOP is highest during the day due to increased aqueous humour secretion and lowest at night due to decreased aqueous humour secretion at night (52). Interestingly these patterns of secretion are similar in both nocturnal (rodents) and diurnal (human) studies. In this study, GSH levels in the aqueous humour mirror the same pattern as fluctuations in IOP which makes sense given that GSH is delivered to the lens via the aqueous humour. This suggests that there is some circadian input into the control of lens GSH levels and that GSH availability to the lens differs at different times of the day. In the rat lens, low GSH levels in the aqueous humour and high levels of GSH in the lens may reflect an increase in the uptake of GSH by the lens, and together with GSH synthesis and/or regeneration of GSH may enable the lens to ensure GSH levels are highest towards the start of night. In terms of diurnal animals like humans, it is expected that GSH levels in the human aqueous humour would mirror that of the rat aqueous humour, but that GSH levels in the human lens would be higher during the day versus the night to counteract increased oxidative stress encountered during the day. However, testing using a diurnal animal model would be required to confirm this.

Taken together, our findings demonstrate the lens to contain the molecular machinery of a circadian clock that may be used to ensure high GSH levels in the lens are available to protect against ROS generated through increased metabolic activity and/or exogenous sources. Further work will be aimed at knocking down the expression of BMAL1 to see the effect this has on the other clock component genes as well as NRF2 and GSH levels. Moreover, while our findings implies that there is a rhythmic expression of clock and redox proteins and GSH levels in the lens, further experiments are required to demonstrate that these rhythmic expressions persist in constant darkness and are therefore truly circadian in nature. This will help to establish a direct link between a circadian clock in the lens regulating GSH levels. Since GSH levels in the lens are known to decline with age, understanding these mechanisms may provide a better understanding as to whether the circadian clock can be used to restore GSH levels in the lens with advancing age.

## Data Availability

The original contributions presented in the study are included in the article/**Supplementary Material**, further inquiries can be directed to the corresponding author/s.

## Supplementary material

Supplementary Figure 1 Expression of negative arm clock component proteins in the rat lens at different times of the day. **(A)** Expression of CRY1, **(B)** PER2 and **(C)** PER3 over a 24-hour time period. Lens tissue (n=8 rats) was harvested at 4-hour intervals over a 24-hour period. 20µg lens protein/lane was electrophoresed on an SDS PAGE gel and protein levels analysed by Western blotting and expressed relative to the β-actin internal control. Antibodies for CRY1 (Protein Tech 13474-1-AP), PER2 (Protein tech 12550-1-AP), PER2 (Abcam, ab179813) and PER3 (Protein Tech 12550-1-AP) were tested. Most western blots revealed no bands or very faint bands with high background with the “best” blots for CRY1, PER2 and PER3 expression shown. While the PER3 blot looks promising, repeated western blots failed to give a similar result making it difficult to obtain a reliable pattern of expression.
