# Supplementary figures and images for: Time of day differences in the regulation of glutathione levels in the rat lens
Source: Front Ophthalmol (Lausanne). 2024 Aug 15;4:1407582. doi: 10.3389/fopht.2024.1407582 (PMC11358124; doi:10.3389/fopht.2024.1407582)

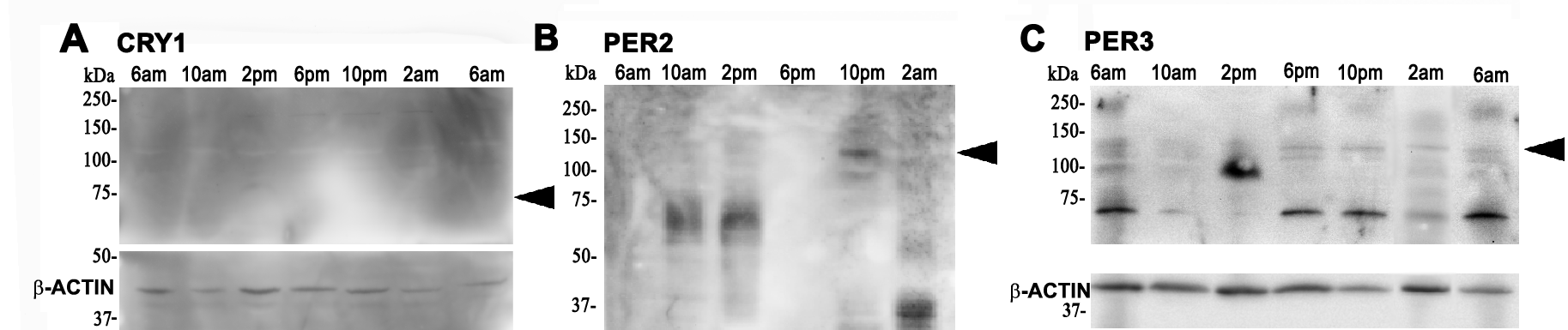

Supplement: Supplementary Figure 1 — Expression of negative arm clock component proteins in the rat lens at different times of the day. (A) Expression of CRY1, (B) PER2 and (C) PER3 over a 24-hour time period. Lens tissue (n=8 rats) was harvested at 4-hour intervals over a 24-hour period. 20µg lens protein/lane was electrophoresed on an SDS PAGE gel and protein levels analysed by Western blotting and expressed relative to the β-actin internal control. Antibodies for CRY1 (Protein Tech 13474-1-AP), PER2 (Protein tech 12550-1-AP), PER2 (Abcam, ab179813) and PER3 (Protein Tech 12550-1-AP) were tested. Most western blots revealed no bands or very faint bands with high background with the “best” blots for CRY1, PER2 and PER3 expression shown. While the PER3 blot looks promising, repeated western blots failed to give a similar result making it difficult to obtain a reliable pattern of expression. [file Image1.tif]
